# Digital engagement in the silver economy

**DOI:** 10.3389/fpubh.2026.1804832

**Published:** 2026-05-05

**Authors:** Ieva Meidute-Kavaliauskiene, Olga Iurasova, Artūras Jakubavičius

**Affiliations:** Business Management Faculty, Vilnius Gediminas Technical University, Vilnius, Lithuania

**Keywords:** association, digital, digital engagement, GDP per capita, people aged 55–74, silver economy

## Abstract

**Background:**

Population aging and accelerating digitalization increase the relevance of the silver economy, as country-level patterns of digital engagement among older age groups shape inclusion in markets and services.

**Methods:**

This study examines the country-level association between productive digital engagement among people aged 55–74 and two macroeconomic outcomes (GDP per capita and GNI per capita) across European countries plus Iceland and Norway, using OECD ICT Access and Usage by Households and Individuals indicators. Independent variables measure the national prevalence of purpose-specific online activities (online purchasing, online course participation, use of online ICT services, social networking, and health information search), a skills barrier (lack of skills for submitting online forms), and a digital infrastructure proxy (fixed broadband subscriptions). A multivariate linear regression model is estimated, and multicollinearity is assessed.

**Results:**

The models explain a substantial share of cross-country variation in GDP per capita (R^2^ ≈ 0.65) and GNI per capita (R^2^ ≈ 0.73). Countries with higher shares of people aged 55–74 engaging in online purchasing tend to exhibit higher GDP and GNI per capita; online purchasing remains a strong positive correlate in both specifications. In contrast, the association of online course participation varies by outcome measure. Health information search and social networking show negative partial associations in both models, while general internet-intensity indicators (rare/daily use) add limited explanatory power due to multicollinearity.

**Conclusion/implications:**

Cross-country differences in GDP per capita align more closely with the country-level prevalence of transactional and skill-building digital activities among people aged 55–74 than with internet-use frequency alone. Because the analysis is observational and based on national aggregates, the findings indicate associations at the country level and should not be interpreted as causal or as individual-level relationships.

## 1 Introduction

The Silver Economy reflects a shift in economic thinking: population aging is increasingly seen not only as a fiscal cost or ‘burden’ issue, but also as a source of significant economic opportunities and new policy challenges. In European Union policy and analytical documents (1–3), the Silver Economy is defined as the totality of all economic activities that meet the needs of the population aged 50 and over, encompassing both the goods and services they directly purchase and the additional economic activity that this consumption creates (value chain effects). Such a definition expands the traditional view of aging. It emphasizes the role of older age groups as consumers, service users, technology adopters, and (in some cases) participants in the labor market and lifelong learning (4–6). For this reason, demographic aging is considered a structural factor in the transformation of the economy and public services in the European context: the Silver Economy is becoming important not only in terms of market development, but also in terms of the accessibility and efficiency of public sector services (e.g., health, social security, mobility, administrative services).

While the Silver Economy is conceptually defined as encompassing the population aged 50 and over, the empirical analysis in this study focuses on the 55–74 age group. This restriction follows the structure of harmonized OECD ICT indicators, which are reported in standard age bands and provide the most consistent cross-country coverage for people aged 55–74 over the study period. Substantively, ages 55–74 represent a core segment of the Silver Economy: many individuals are still economically active or recently retired, increasingly interact with digitalized public and private services, and display substantial cross-country variation in both digital skills and purpose-specific online behaviors. By contrast, ICT-use patterns among those aged 75 + are more strongly shaped by health limitations and dependency and are less consistently measured in internationally comparable datasets. Accordingly, the findings should be interpreted as evidence on productive digital engagement within the Silver Economy, specifically for the 55–74 subpopulation, while acknowledging limited generalizability to the oldest-old without additional data.

Within this framework, the role of digital transformation is particularly evident. EU-level analyses highlight those digital solutions (remote services, e-commerce, e-health, digital identity tools, etc.) can support the independence of older people, increase social and economic inclusion, and improve the efficiency of service provision. Digitalization in the context of the Silver Economy is usually associated with two interrelated mechanisms: first, technologies can reduce transaction costs and improve the accessibility and efficiency of services, as digital channels allow for faster procedures and reduced administrative burdens (e.g., e-services, e-billing, e-commerce); second, they can strengthen human capital through lifelong learning, skills upgrading and broader opportunities for social inclusion. It is this logic that allows us to move from the general question of whether older populations are online to a more economically informative question: for what purposes they use the Internet and whether that use is ‘productive’, i.e., related to learning, services, and market participation.

This article aims to assess how productive digital engagement (e.g., online shopping, participation in distance learning courses, use of online ICT services) and digital skills barriers in in EU countries plus Iceland and Norway are related to economic well-being, measured by GDP per capita. To achieve this goal, the article: (1) theoretically substantiates the link between the silver economy and productive digital use with economic development; (2) provides descriptive statistics and an analysis of country heterogeneity; (3) empirically assesses the relationships between purpose-specific online activity prevalence among people aged 55–74 and GDP/GNI per capita, distinguishing the importance of “productive” activities compared to the intensity of digital technology use; (4) constructs and evaluates a multivariate regression model, identifying statistically significant factors and the direction of their associations with GDP/GNI per capita; (5) performs model diagnostics (error structure, influence points, forecasting errors, multicollinearity) and discusses the limits of interpretation; (6) formulates practical insights for policy measures aimed at strengthening the digital skills of older people and increasing their inclusion in economically meaningful digital activities.

The remainder of the article is structured as follows. First, the literature review conceptually substantiates the significance of the Silver Economy and productive digital engagement (e-commerce, distance learning, e-services) for economic development, emphasizing the role of the digital divide and digital skills. Second, the methodology section describes the data, variables, and empirical strategy. Third, the results section reports the empirical associations between people aged 55–74 ICT-use indicators and GDP per capita and presents model diagnostics. Finally, the discussion and conclusion summarize theoretical and practical implications and outline policy interventions most relevant for increasing people aged 55–74 inclusion in economically meaningful digital activities and reducing the digital divide.

## 2 Literature review

Population aging and the accelerating digital transformation are two major development trajectories of modern economies, fundamentally reconfiguring the structure of consumption, the provision of public services, the labor market, and social welfare systems. At the intersection of these phenomena, the “Silver Economy” is taking shape, a concept that reflects a paradigm shift in economic thinking: aging is increasingly seen not only as a problem of increasing costs and dependency, but also as a space for potential markets, innovations, and new policy solutions (7). The “Silver Economy” encompasses the totality of all economic activities, products and services designed to meet the needs of the older population, while recognizing older persons as active economic participants—consumers, service users, technology adopters and, in some instances, participants in the labor market and lifelong learning (5, 6). This approach is particularly relevant in the European context, where demographic aging is a structural driver of economic and public-service transformation. Accordingly, the senior segment matters not only for market development, but also for the accessibility and efficiency of key services, including health, social security, mobility, and public administration.

The modern Silver Economy is increasingly being analyzed through the prism of active aging, emphasizing that the well-being and economic role of older people depend on maintaining their independence, social inclusion, and opportunities to participate in economic life. As Przybysz and Stanimir (6) emphasize, the Silver Economy encompasses various development opportunities, including ensuring long-term professional activity, maintaining independence, optimizing time use, meeting health and personal image needs, fostering social inclusion, and creating age-sensitive financial services. These dimensions constitute not only a set of “services for seniors”, but also a broader economic ecosystem in which the public sector and businesses must combine innovation implementation with social and institutional constraints. Heffner et al. (8) emphasize that the Silver Economy is a multidimensional construct that enables the exploitation of opportunities provided by aging while reducing its adverse consequences. Still, these strategies require a consistent assessment of determinants and barriers—especially those related to technological literacy and digital engagement. This is why the Silver Economy is increasingly conceptualized today in the “digital age,” when a large part of economic and social activities is moving to digital platforms, and public and private service ecosystems are becoming “digital-first.” The digital economy is described as a structural transformation driven by new generation technologies—mobile internet, cloud computing, big data, and artificial intelligence that have become engines of productivity and growth (9). However, this transformation also creates risks of inequality, as the benefits of the digital economy are unevenly distributed both within and between countries. Bukht and Heeks (10) distinguish two key risks associated with digital exclusion: first, outright exclusion from digital opportunities due to limited access or skills; second, “adverse incorporation,” where vulnerable groups enter the digital economy under unequal conditions because of weak resources, institutions, and social relations (liminality). This logic is particularly important for the older population, as digital transformation can be both a means of empowerment and a new mechanism of exclusion (11).

Digital skills and digital engagement define the extent to which older people can benefit from digitalized services and markets, and the extent to which they can contribute to economically mediated systems. In other words, the key issue is not internet use per se, but the purposes for which people aged 55–74 go online and whether these uses generate tangible economic and social benefits. In this context, it is increasingly proposed to distinguish fundamental digital inclusion (access, basic use) from meaningful, economically “embedded” participation—productive digital engagement, encompassing e-services, e-commerce, distance learning, digital finance, and other transactional activities. However, as research shows, the path to such engagement is often limited by the digital divide, which is multidimensional and encompasses not only the availability of technologies but also the ability to use them, trust, motivation, perceptions of security, and social support. Bunyan and Collins (12) found that digital exclusion is statistically significantly associated with socioeconomic factors: lower-income individuals, older age groups, and those with lower levels of education are more likely to experience digital exclusion. This insight is essential in the context of the Silver Economy, as limited digital participation can mean not only individual inconvenience, but also wider economic consequences, as Adamczyk and Betlej (13) argue, for example, that digital exclusion in aging societies has systemic social and economic consequences, and technological changes that are not adapted to social needs can promote negative phenomena, including unsustainable economic development. Thus, digital exclusion in old age becomes not only a matter of “social policy” but also a topic of economic productivity, service efficiency, and inequality management. Solomon and Klyton (14) and Shibambu (15) highlight that digital divide issues are particularly pronounced in regions where the overall level of digitalization and infrastructure is weaker, and, as a result, some countries are not achieving the expected level of economic digitalization due to persistent digital gaps: digital skills gaps, ICT infrastructure deficits, and high-cost structures. This is important for the global Silver Economy, as older age groups in these contexts face a “double risk”—both due to age and weaker systemic conditions (16).

One crucial aspect of the intersection of the Silver Economy and digital skills is health and e-health literacy. Digital health services, remote consultations, online health information searches, and electronic systems can increase accessibility and independence, but they also require digital skills (17). Show that low-income seniors (especially those living at home) use the Internet significantly less often than the general population due to limited computer and Internet access, limited financial resources, and health and disability limitations. Li and Yang (18) add that digital literacy has a significant impact on health outcomes, and the strength of the effect varies depending on age, cultural context, and income; their analysis identifies the mechanisms through which digital literacy improves health: better access to information, social networks, medical accessibility, and planned behavior pathways. These results suggest that increasing digital skills can have a “double dividend”—better health and well-being outcomes and greater economic participation. Studies by You et al. (19), Zhang et al. (20), and Zhao & Li (21) on the impact of the digital economy on health inequalities show that its development can reduce health inequalities. Still, the effects are uneven across regions and age groups, and therefore targeted measures for the older segment are necessary.

The issue of digital skills in the context of the Silver Economy extends beyond basic technical literacy (22, 23). Digital literacy is commonly conceptualized as a multidimensional competence, combining technical skills with the ability to apply digital technologies meaningfully in everyday life, work, learning, and social participation (24). At the same time, the literature notes the ambiguity of terminology: “digital skills,” “digital literacy,” and “digital competences” are often used as synonyms, although they have different meanings and emphases. This conceptual ambiguity is necessary for empirical research because the choice of indicators (e.g., skill barriers to e-forms, use of e-services, e-commerce, distance learning) determines how digital inclusion is interpreted and through what channels it is associated with economic well-being. At the same time, it is necessary to recognize that progress in the digital economy does not always automatically translate into productivity growth. Watanabe et al. (25), Meng and Wen (26), and Rybaczewska and Sparks (27), analyzing the “productivity paradox,” raised the question of under what conditions technological progress is translated into tangible economic value. In the Silver Economy, this paradox may be even more pronounced if older individuals lack sufficient skills in using digital tools and limit their digital behavior to less transactional activities. Wang (24) emphasizes that the “content” and “quality” of digital use are becoming increasingly important: productive digital engagement (online shopping, learning, services) may be more related to economic well-being than the frequency of use alone. This argument is also strengthened by evidence that the effects of the digital economy (e.g., on subjective well-being or happiness) are heterogeneous by age and by the intensity of use (21), suggesting the need to differentiate interventions by age segments and types of digital participation.

Against the backdrop of these challenges, digital inclusion strategies take on particular importance. Policy interventions aimed at reducing the digital divide must include not only the availability of devices and connectivity, but also targeted measures to develop competencies, build trust, and provide support infrastructure (e.g., counseling, mediation). Li (21) identifies several channels through which the digital economy can impact well-being—the promotion of digital applications, improving information literacy, and optimizing the digital living environment—and shows that these impacts operate through economic improvement, health promotion, quality of life, environmental optimization, and governance efficiency. This is important for Silver Economy policies because it indicates specific “leverages” through which digital tools can create benefits for older people (17, 28). In addition, evidence suggests that e-government development is associated with broader digital economy advancement and can function as a platform for improving citizens’ digital engagement, including among people aged 55–74 (29). However, it is also necessary to consider issues of technology design, for example, Neven (30) raises the question of whether technological solutions actually meet the needs of older people or only “adapt” them to the system; too “passive” adaptation of technologies can limit the meaningful engagement of older people. This means that digital inclusion must be understood not only as technical training but also as a broader environment, including interfaces, service design, and social support (13, 31).

Although prior research provides extensive evidence on the Silver Economy, digital inclusion, and the digital divide among a people aged 55–74, it remains insufficiently clear which specific types of online engagement in later life are most closely aligned with macro-level economic prosperity. Much of the literature focuses on access, general internet use, or skills barriers. It often examines single domains (e.g., e-health literacy or social participation) in isolation. At the same time, ICT-growth studies typically emphasize infrastructure or aggregate ICT measures rather than the behavioral profile of people aged 55–74. Consequently, it remains unresolved whether transactional and skill-building activities (e-commerce, online learning, and online service use) differ systematically from other common online behaviors (social networking and health information searching) in their association with GDP per capita once multiple ICT dimensions are considered jointly. This study advances the literature by using harmonized OECD indicators for ages 55–74 to operationalize productive digital engagement, contrasting it with general intensity measures, and linking these purpose-specific behaviors to GDP per capita in an EU panel setting, complemented with diagnostics for influential observations and multicollinearity.

Accordingly, the following section outlines the dataset and the empirical approach to quantify the association between distinct forms of digital engagement among people aged 55–74 (at the country level) and GDP/GNI per capita across EU countries.

## 3 Methodology

The empirical analysis uses annual country-level panel data for European Union countries and Iceland and Norway from 2011 to 2024. Indicators describing ICT access and usage among people aged 55–74 were obtained from the OECD ICT Access and Usage by Households and Individuals database, focusing on the 55–74 age group.

This research used the following variables:

- Lack of skills for submitting forms.

Measures the share of individuals who did not submit forms to public authorities online during the last 12 months due to a lack of skills or knowledge. It reflects digital competency barriers in relation to e-government service usage (percentage of population) (32).

- Individuals using the Internet (last 12 months).

Indicates the proportion of individuals who used the Internet at least once during the past 12 months. It functions as a broad indicator of baseline digital inclusion and connectivity among people aged 55–74 (percentage of population; OECD Going Digital Toolkit) (33).

- Fixed broadband subscriptions.

Captures the total number of fixed broadband Internet subscriptions in a country. While not age-specific, the indicator serves as a structural proxy for national digital infrastructure and service availability, enabling households to access the internet (mln. of subscriptions) OECD Going Digital Toolkit (34).

- Daily Internet use (last 3 months).

Represents the share of individuals who used the Internet daily or almost daily in the preceding 3 months. This variable distinguishes between frequent and occasional users, reflecting the intensity of their digital engagement (percentage of population) (35).

- Social networking use (last 3 months).

Measures the proportion of individuals who used the Internet to access social networking platforms during the last 3 months. It is a behavioral indicator related to communication, social participation, and digital social integration (percentage of population) (36).

- Online course participation (last 3 months).

Denotes the share of individuals (55–74 years) who took an online course in any subject in the preceding 3 months. It serves as a proxy for lifelong learning, skill development, and digitalization of education among people aged 55–74 (percentage of population) (37).

- Seeking health information online (last 3 months).

Indicates the proportion of individuals (55–74 years) who sought health-related information online in the past 3 months. This behavior is linked to digital health literacy and the integration of digital tools into personal healthcare management (percentage of population) (38).

- Online purchasing (last 12 months).

Measures the share of individuals (55–74 years) who purchased goods or services online within the past 12 months. It reflects digital market participation, consumer readiness, and trust in e-commerce environments (percentage of population) (39).

As the dependent variables were chosen:

- GDP per capita—annual value of finished goods and services for country i in year t, measured in current US$.
- GNI per capita—gross national income per person, measured in current US$.

The dependent variables are GDP per capita and GNI per capita (current US$) (40). The final dataset consists of 27 EU countries and Iceland and Norway, which are members of the European Economic Area (EEA) and EFTA yielding up to 378 country-year observations. Observations with missing values in the dependent variable or key regressors were excluded (Malta, Cyprus) to obtain a balanced set of country-year records for estimation and diagnostics. The final dataset used for estimation contains 278 observations.

Next, a normalization algorithm was applied to mitigate errors arising from different measurement scales (41). Minimum-maximum normalization with a 0–1 scale shows good effectiveness for next regression analysis (42).

The empirical relationship between each dependent variable and ICT-related predictors was estimated using a pooled panel linear regression framework applied to the full country-year dataset for European countries over 2011–2024 (Equation 1):


$$y_{\mathrm{it}}=\alpha +\beta X_{\mathrm{it}}+\varepsilon_{\mathrm{it}}$$ (1)

Where:

*α* is the intercept,

*β* is the vector of slope coefficients,

$\varepsilon_{\mathrm{it}}\;$ is the idiosyncratic error term.

A pooled panel regression approach was adopted as a baseline specification to capture cross-country variation, with robustness checks considered through alternative specifications (exclusion of highly collinear variables). Coefficients are interpreted as conditional associations between the ICT indicators and each dependent variable, holding other variables constant (43). Given the cross-country panel structure and the likelihood of heteroskedasticity, inference is reported using heteroskedasticity-robust standard errors; where appropriate, errors may be clustered by country to account for within-country serial correlation.

The main estimation step consists of fitting the linear model to the full panel dataset and reporting coefficient estimates, standard errors, t-statistics, and *p*-values for P > |t|. Model explanatory power is summarized using R2 and adjusted R2. This step provides the primary evidence on which ICT dimensions are most strongly associated with GDP per capita in the EU context.

After estimating the linear regression model, its predictive performance was evaluated using year-level accuracy metrics. This step complements the inferential analysis by assessing how well the estimated model reproduces observed GDP per capita values across time (44).

Year-level accuracy metrics were computed for 2020–2023. For each year t, predicted values $\hat{y_{\mathrm{it}}}$ were generated and compared with the observed GDP per capita $\hat{y_{\mathrm{it}}}\;$ .

The comparison was conducted by aggregating country-level observations within each year, allowing for the assessment of temporal prediction accuracy and the model’s stability across different periods.

The following accuracy metrics were computed:

- Mean (actual and predicted values). For each year, the mean of observed GDP per capita and the mean of predicted values were calculated. This provides a baseline comparison of central tendency, allowing identification of systematic over- or underestimation at the aggregate level (45).
- Bias (Predicted−Actual). Bias was computed as the difference between predicted and observed values. This metric captures systematic prediction error, indicating whether the model consistently overestimates (positive bias) or underestimates (negative bias) GDP per capita (45).
- Mean Absolute Error (MAE). MAE measures the average magnitude of prediction errors in absolute terms. It reflects the average deviation between predicted and actual values, providing an interpretable measure of prediction accuracy that is not sensitive to the direction of errors (46).
- Root Mean Squared Error (RMSE). RMSE was calculated to capture the square root of the average squared prediction errors. Compared to MAE, RMSE penalizes larger deviations more strongly, making it particularly useful for detecting large prediction errors or outliers (46).
- Correlation (actual vs. predicted values). The Pearson correlation coefficient between observed and predicted GDP per capita values was computed for each year. This metric evaluates the strength and direction of the linear relationship between predictions and actual values, indicating how well the model captures the relative variation across countries (47).

Together, these metrics provide a comprehensive evaluation of model performance, capturing central tendency alignment (mean), systematic error (bias), average deviation (MAE), sensitivity to large errors (RMSE), and structural consistency (correlation). This multidimensional assessment rigorously evaluates predictive accuracy and the stability of the estimated relationships over time. To identify country-year observations exerting disproportionate influence on coefficient estimates, Cook’s distance was computed for each observation (48) (Equation 2):


$$D_{i}=\frac{\sum_{j=1}^{n}{\left({\hat{y}}_{j}-{\hat{y}}_{j(i)}\right)}^{2}}{p\cdot MSE}$$ (2)

where ${\hat{y}}_{j(i)}$ denotes the fitted value for observation j when observation i is omitted,

p is the number of estimated parameters,

MSE is the mean squared error of the model.

Following the research design, observations with Cook’s distance exceeding the predefined threshold of 0.01 were classified as influential. This procedure enables assessment of whether a small number of structurally atypical economies disproportionately drive estimated relationships. These influential points represent cases in which the relationship between people aged 55–74 ICT-use indicators and GDP per capita deviates materially from the typical EU pattern, thereby shifting estimated elasticities and even coefficient signs.

Given the conceptual overlap among ICT usage indicators, multicollinearity was assessed using the variance inflation factor (VIF) computed for each regressor (Equation 3):


$$\mathrm{VI}F_{k}=\frac{1}{1-R_{k}^{2}}$$ (3)

Where $R_{k}^{2}$ - the coefficient of determination from regressing predictor k on all other predictors? A VIF threshold of 10 was applied to identify severe multicollinearity (49). Variables exceeding this threshold were flagged as potentially redundant, indicating that coefficient estimates may be unstable and standard errors inflated. This diagnostic informed robustness considerations regarding alternative specifications (e.g., excluding redundant intensity measures or using composite indices) to improve interpretability and stability.

The combined workflow of estimation, accuracy evaluation, influence diagnostics, and multicollinearity testing provides a structured analytical pipeline. Regression coefficients provide inferential evidence on associations with GDP per capita, while the accuracy metrics evaluate predictive coherence; Cook’s distance identifies leverage points that may distort inference; and VIF results quantify redundancy among predictors.

To assess whether the baseline regression results are robust to persistent cross-country differences that are not directly observed in the dataset, an additional panel specification with country fixed effects was estimated. This robustness step is important because the empirical sample consists of repeated annual observations for the same countries, and unobserved time-invariant national characteristics may affect GDP per capita as well as ICT-related behavior among people aged 55–74.

The fixed-effects calculation demonstrates each country time-invariant country-specific effects. As a result, the estimated coefficients are identified from within-country variation over time and show whether changes in the country-level prevalence of digital behaviors among people aged 55–74 within the same country are associated with changes in GDP per capita. In this sense, the fixed-effects model provides a more restrictive and methodologically stronger test of the baseline relationships.

The robustness check was implemented by estimating the regression model with country fixed variables. The same dependent variable and ICT-related regressors were retained, allowing direct comparison with the baseline pooled model. Statistical significance, coefficient magnitude, and sign stability were then compared across the two specifications. If a coefficient remained statistically significant after the inclusion of country fixed effects, the relationship was interpreted as robust to unobserved time-invariant heterogeneity. If a coefficient lost significance, this was interpreted as evidence that the original association was driven primarily by persistent cross-country differences rather than within-country temporal changes.

This robustness step strengthens the methodological validity of the study by distinguishing between broad structural differences across countries and more stable within-country relationships over time.

All steps were implemented consistently across the panel to ensure transparency and replicability, and results are reported with sufficient detail to allow other researchers to replicate them.

The methodological contribution of this study lies in integrating age-specific digital engagement indicators into a macroeconomic panel framework, combining behavioral (usage), structural (infrastructure), and skills-based variables in a unified empirical model. Unlike prior studies that typically focus either on aggregate digitalization indices or single ICT dimensions, this approach allows for disentangling multiple channels of digital engagement within the silver economy and assessing their differentiated association with economic performance. Additionally, the study incorporates a structured diagnostic pipeline (influence analysis, multicollinearity testing, and predictive validation), which extends beyond standard regression applications in this field.

## 4 Results

This section begins with an exploratory analysis of the dataset and evaluates whether people aged 55–74 ICT-use indicators are associated with GDP per capita across EU countries. Descriptive statistics indicate substantial heterogeneity across countries in both older adults internet-related behaviors and economic development (Table 1). “Lack of skills for submitting forms” ranges from almost negligible levels (0.040) to 16.714, with a mean of 4.450 and a moderate standard deviation of 3.179 (variance 10.104). The positive skewness (1.146) and kurtosis (1.322) show a right-skewed, slightly leptokurtic distribution: in most countries, the lack-of-skills barrier is relatively low, while a smaller group exhibits considerably higher values.

**Table 1:** Description statistics.

Column	Min	Max	Mean	Std. deviation	Variance	Skewness	Kurtosis
Lack of skills for submitting forms	0.040	16.714	4.450	3.179	10.104	1.146	1.322
Rare using	21.204	98.023	61.890	20.957	439.184	0.023	−1.124
Subscriptions Fixed broadband	0.0001	35.191	2.631	6.242	38.963	3.683	13.611
Daily using	8.719	91.642	45.586	20.837	434.166	0.391	−0.907
Social networking	5.448	81.677	25.486	14.824	219.757	1.276	1.796
Online course	0.217	10.070	2.658	2.076	4.310	1.315	1.329
Seeking health information	11.889	71.753	36.038	12.977	168.405	0.269	−0.357
Online purchasing	2.117	72.561	29.250	19.997	399.877	0.447	−1.091
GDP per capita	7.268	137.781	39.746	27.307	7457.724	1.385	1.756

“Subscriptions Fixed broadband” exhibits by far the largest dispersion. Values range from 18.550 to 35,191,417.000, with a mean of 2,631,645.216 and a very large standard deviation of 6,242,045.920 (variance 38,963,137,266,528.700). The high positive skewness (3.683) and kurtosis (13.611) indicate a highly right-skewed, strongly leptokurtic distribution, suggesting that a few countries have extremely high subscription counts relative to the rest of the sample.

The indicator of rare internet use spans from 21.204 to 98.023, with an average of 61.890 and a standard deviation of 20.957 (variance 439.184). Skewness is essentially zero (0.023), indicating an approximately symmetric distribution around the mean. In contrast, negative kurtosis (−1.124) suggests a flatter-than-normal (platykurtic) distribution, with fewer extreme values than a Gaussian distribution.

The indicator for Daily use of the Internet ranges from 8.719 to 91.642, with an average of 45.586 and a standard deviation of 20.837 (variance 434.166). Skewness is mildly positive (0.391), indicating a slight concentration of countries below the mean and a longer right tail, while kurtosis is negative (−0.907), again suggesting a relatively flat distribution with fewer extreme outliers.

The share of people using social networks varies from 5.448 to 81.677, with a mean of 25.486 and a standard deviation of 14.824 (variance 219.757). The skewness value of 1.276 and the kurtosis of 1.796 indicate a clearly right-skewed, moderately leptokurtic distribution, with many countries clustered at lower participation levels and a smaller subset showing notably high engagement in social networking.

Participation in Online courses is comparatively low, ranging from 0.217 to 10.070. The mean is 2.658, and the standard deviation is 2.076 (variance 4.310). The distribution is right-skewed (skewness 1.315) and slightly leptokurtic (kurtosis 1.329), implying that most countries have modest levels of online course use among people aged 55–74, while a few countries achieve substantially higher rates.

The share of the population seeking health information online ranges from 11.889 to 71.753, with an average of 36.038 and a standard deviation of 12.977 (variance of 168.405). Skewness is small and positive (0.269), indicating only slight right skew, whereas kurtosis is mildly negative (−0.357), consistent with a near-normal but somewhat flatter distribution.

The share of the population engaging in online purchasing ranges from 2.117 to 72.561, with a mean of 29.250 and a standard deviation of 19.997 (variance of 399.877). Skewness is moderately positive (0.447), indicating that most countries lie below the mean and have a longer upper tail. Kurtosis is negative (−1.091), reflecting a platykurtic distribution with relatively fewer extreme values.

GDP per capita ranges widely from 7,268.654 to 137781.6817, with a mean of 39746.286 and a high standard deviation of 27307.964. The positive skewness (1.261) and kurtosis (1.351) indicate a right-skewed, moderately leptokurtic distribution, with a few high-income countries pulling the distribution’s right tail upward. Overall, the statistics highlight pronounced cross-country disparities in both digital engagement of people aged 55–74 and economic prosperity, with several variables displaying non-normal distributions characterized by right skewness and, in some cases, heavy tails.

Using the described variables, the Linear Regression Model was created. The model demonstrates high explanatory capacity (R^2^ = 0.6479; Adjusted R^2^ = 0.6415) and indicators jointly explain roughly 74–75% of the cross-sectional and temporal variation in GDP per capita across EU countries (Table 2).

**Table 2:** Linear regression results.

Variable	Coefficients	
	Dependent variable GDP per capita	Dependent variable GNI per capita
Lack of skills for submitting forms	0.208 (0.000)*	0.1579 (0.005)*
Rare using	0.0357 (0.752)****	−0.0469 (0.783)****
Subscriptions Fixed broadband	−0.1983 (0.000)*	−0.148 (0.037)**
Daily using	0.2102 (0.153)****	0.4144 (0.068)***
Social networking	−0.221 (0.00)*	−0.3431 (0.000)*
Online course	0.447 (0.00)*	0.0002 (0.998)****
Online ICT services	0.1115 (0.050)**	0.0841 (0.365)****
Seeking health information	−0.461 (0.000)*	−0.4247 (0.000)*
Online purchasing	0.770 (0.000)*	0.992 (0.000)*
Intercept	0.035 (0.078)	0.1287 (0.002)*

*Statistically significant at 1% level. **Statistically significant at 5% level. ***Statistically significant at 10% level. ****Statistically unsignificant.

The estimated associations with GDP per capita are as follows: The largest and most statistically robust positive coefficient in both models is for online purchasing. In the GDP per capita model, its coefficient is *β* = 0.770, *p* = 0.000, while in the GNI per capita model it becomes even larger, *β* = 0.992, *p* = 0.000. This indicates that countries with a higher share of purchasing online tend to have both higher GDP per capita and higher GNI per capita E-commerce adoption among people aged 55–74 appears to be a strong marker of digital-market maturity at the country level. The stronger coefficient in the GNI model suggests that this type of digital market participation is associated not only with domestic output, but even more strongly with resident-income-based development.

A major difference between the two models appears for online course participation. In the GDP model, this variable has a strong positive and highly significant coefficient (*β* = 0.447, *p* = 0.000), indicating that greater participation in online courses among people aged 55–74is associated with higher GDP per capita. This supports a human capital / lifelong learning interpretation, whereby wealthier economies tend to have stronger adult learning systems, better digital skills, and greater institutional support for reskilling. However, in the GNI model this relationship disappears completely (*β* = 0.0002, *p* = 0.998). This suggests that the positive association between online learning and macroeconomic development is not robust to the use of GNI per capita and may be more closely linked to production-based prosperity than to the income retained by residents.

The variable Lack of skills for submitting forms remains positive and statistically significant in both models, although it is somewhat weaker for GNI. In the GDP model, the coefficient is *β* = 0.208, *p* = 0.000; in the GNI model it is *β* = 0.1579, *p* = 0.005. As in the GDP interpretation, this positive sign should not be read as meaning that lower skills increase prosperity. Rather, in higher-income and more digitalized countries, people aged 55–74 are more frequently exposed to e-government systems and therefore are more likely to report lack-of-skills barriers explicitly. In lower-income countries, non-use of e-government may reflect weaker service availability rather than a consciously reported skill deficit. The fact that the coefficient remains significant in both models suggests that this variable captures the broader institutional penetration of digital public services rather than a direct productivity effect.

The variable Seeking health information online shows a strong negative and highly significant association in both models. For GDP per capita, the coefficient is *β* = −0.461, *p* = 0.000; for GNI per capita, it is *β* = −0.4247, *p* = 0.000. A higher share of people aged 55–74 searching for health information online is associated with lower levels of macroeconomic development, regardless of whether this is measured by domestic output or resident income. This may reflect need-driven behavior, where individuals in lower-income or less accessible healthcare systems rely more on online information as a substitute, or it may proxy an aging-related burden that remains after controlling for more economically embedded digital behaviors.

The coefficient for Subscriptions Fixed broadband is negative and statistically significant in both models, but it is somewhat weaker in the GNI specification. In the GDP model, the coefficient is *β* = −0.1983, *p* = 0.000; in the GNI model, *β* = −0.148, *p* = 0.037.

Social networking is also negative and highly significant in both models, and the coefficient becomes even more negative when GNI per capita is used. In the GDP model, *β* = −0.221, *p* = 0.000; in the GNI model, *β* = −0.3431, *p* = 0.000. The stronger negative coefficient in the GNI model may indicate that social-network-oriented internet use is even less closely aligned with resident-income-based development than with GDP-based production outcomes.

The effect of Online ICT services differs more clearly across the two models. In the GDP per capita model, the coefficient is *β* = 0.1115, *p* = 0.050, indicating a weak but statistically significant positive relationship. Service-oriented digital engagement among people aged 55–74 reflects functional digital inclusion, such as using e-government or formal online services that reduce transaction costs and improve efficiency.

The variable Daily using is non-significant in the GDP model (*β* = 0.2102, *p* = 0.153) but becomes much stronger and marginally significant in the GNI model (*β* = 0.4144, *p* = 0.068). The informational content of general internet-use frequency differs depending on which macroeconomic outcome is used.

Rare using remains non-significant in both models: *β* = 0.0357, *p* = 0.752 for GDP per capita and *β* = −0.0469, *p* = 0.783 for GNI per capita. This confirms that occasional internet use does not differentiate development levels once more detailed digital-behavior variables are included in the model. This result is consistent with the earlier multicollinearity findings and supports the argument that general internet-use frequency adds little explanatory power beyond purpose-specific digital engagement.

The intercept is not substantively central in either model, although it is statistically significant in the GNI specification (*β* = 0.1287, *p* = 0.002) and not in the GDP model (*β* = 0.035, *p* = 0.078). It has limited practical meaning, especially if the variables are standardized or centered, and should not be emphasized in substantive discussion.

Table 3 provides year-level accuracy metrics for 2020–2023 and evaluates how well the ICT-based regression approximates cross-country variation in GDP per capita during a period marked by major macroeconomic shocks.

**Table 3:** 2020–2023 year-level accuracy.

Year	N	Mean(actual)	Mean(pred)	Bias (Pred−Actual)	MAE	RMSE	Correlation (actual, prediction)
2020	24	0.2336	0.2476	+0.0140	0.0715	0.0945	0.9076
2021	26	0.2854	0.3044	+0.0190	0.0912	0.1195	0.8628
2022	25	0.2798	0.3197	+0.0399	0.1277	0.1597	0.7376
2023	26	0.2898	0.3059	+0.0160	0.1015	0.1353	0.7944

The evaluation of predictive accuracy by year is restricted to the 2020–2023 period. This design reflects a deliberate separation between model estimation and out-of-sample validation.

The selected evaluation window represents the most recent and policy-relevant period, capturing structural changes in digital engagement among people aged 55–74.

Evaluation to 2020–2023 enables a out-of-sample validation framework within a finite panel setting. While 2011–2019 and 2024 years were used for model creation.

The aim of the evaluation is to verify whether the estimated model provides reliable prediction in the most recent and economically relevant timeframe. The evaluation period over 2020–2023 aligns of current digital engagement dynamics in the silver economy.

Excluding the 2024 year ensures consistency of the evaluation framework, as the 2020–2023 period represents the latest interval with fully harmonized and validated data across all countries and variables included in the model. This allows for a fair and methodologically sound comparison between predicted and observed values.

From a methodological perspective, omitting the last year aligns with common practice in empirical research, where the final observation period is often excluded from validation until data revisions are finalized, ensuring that results are based on stable and reproducible inputs.

The results demonstrate a consistently positive bias (Pred−Actual) across all years, indicating systematic overprediction of GDP per capita. This suggests that, conditional on the observed ICT usage profile, the model expects slightly higher income levels than are realized. Contemporaneous digital participation indicators embed “structural development” signals that did not fully translate into measured GDP per capita during these years; possible reasons include pandemic disruptions, supply constraints, inflationary pressures, and energy-price shocks affecting output and real income dynamics.

The strong correlations in 2020 (0.9076) and 2021 (0.8628) indicate that the model retains strong predictive power: countries with higher GDP per capita are generally predicted to have higher digital engagement, consistent with a stable cross-sectional association between people aged 55–74′ digital engagement and economic prosperity. But predictive precision decreases in 2022, with the correlation falling to 0.7376 and both MAE (0.1277) and RMSE (0.1597) peaking, accompanied by the largest bias (+0.0399). This pattern is economically consistent with a structural break in 2022, when EU economies were unevenly exposed to the energy crisis, terms-of-trade shocks, and sector-specific contractions/expansions. In such conditions, GDP per capita becomes more strongly driven by short-run macro factors not directly captured by household ICT-use variables, weakening the contemporaneous mapping from digital behavior to income.

In 2023, performance partially recovers (correlation 0.7944; MAE 0.1015; RMSE 0.1353), suggesting realignment toward a more typical relationship between digital engagement and economic outcomes as economies adjusted. Overall, these findings imply that the model is effective at explaining medium-run cross-country income differences but less reliable in shock-dominated years.

**Figure 1:**
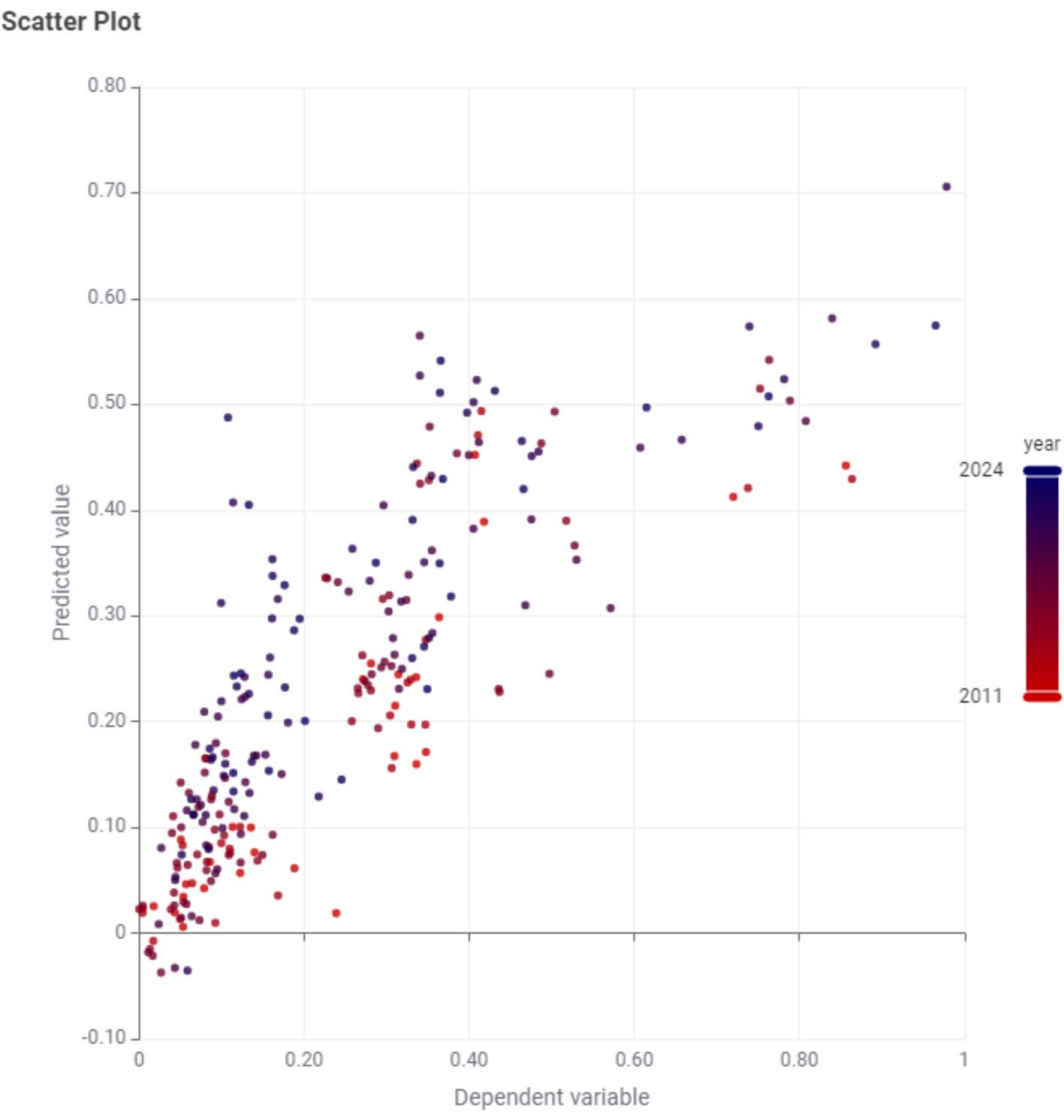
Dependent variable vs. predicted variable.

The ICT-usage indicators among ages 55–74 capture meaningful cross-country variation in GDP per capita, but the model exhibits systematic bias for structurally atypical economies and for countries where digital inclusion does not translate into commensurate productivity/income levels (Figure 1).

Based on the Prediction vs. the Dependent variable few patterns appeared (Table 4):

**Table 4:** Linear regression models’ underprediction by country.

Year	Luxembourg (the largest and most consistent underprediction)	Ireland (consistent underprediction, especially 2022–2023)	Norway (consistent underprediction)
2020	−0.258	−0.148	−0.158
2021	−0.273	−0.166	−0.191
2022	−0.335	−0.271	−0.258
2023	−0.391	−0.255	−0.118

Countries the model systematically underpredicts (Actual > Predicted).

These are typically high-GDP-per-capita observations, where the model “pulls” predictions toward the middle (regression-to-the-mean behavior).

The model fails to capture Luxembourg’s structural “outlier” GDP level (the financial sector, cross-border commuting effects, etc.), so it compresses it downward each year (Table 4).

Ireland’s GDP per capita is well-known to be distorted by multinational accounting effects; a model driven by older adults ICT usage can easily underpredict it.

Countries the model systematically overpredicts (Predicted > Actual).

These are lower-GDP-per-capita observations where predictions are pushed upward toward the mean. The model interprets these countries’ older adults digital participation as higher than it actually is, which often indicates missing macro controls (productivity structure, capital intensity, sector mix) or that “digital inclusion” is not the binding constraint on GDP in those cases.

Higher GDP prediction level than observed, suggesting either.

GDP lags behind digital inclusion metrics (Slovak Republic, Latvia),

The country’s economic structure/productivity does not translate ICT adoption into income at the same rate (Belgium, Sweden),

Some predictor values are unusually high relative to GDP (Estonia).

Few countries have a negative prediction [like Romania in 2021 has (−0.035)], while actual GDP per capita cannot be negative. That just indicates that the dependent variable was normalized, and the linear model is unconstrained (so it can produce values below 0).

**Figure 2:**
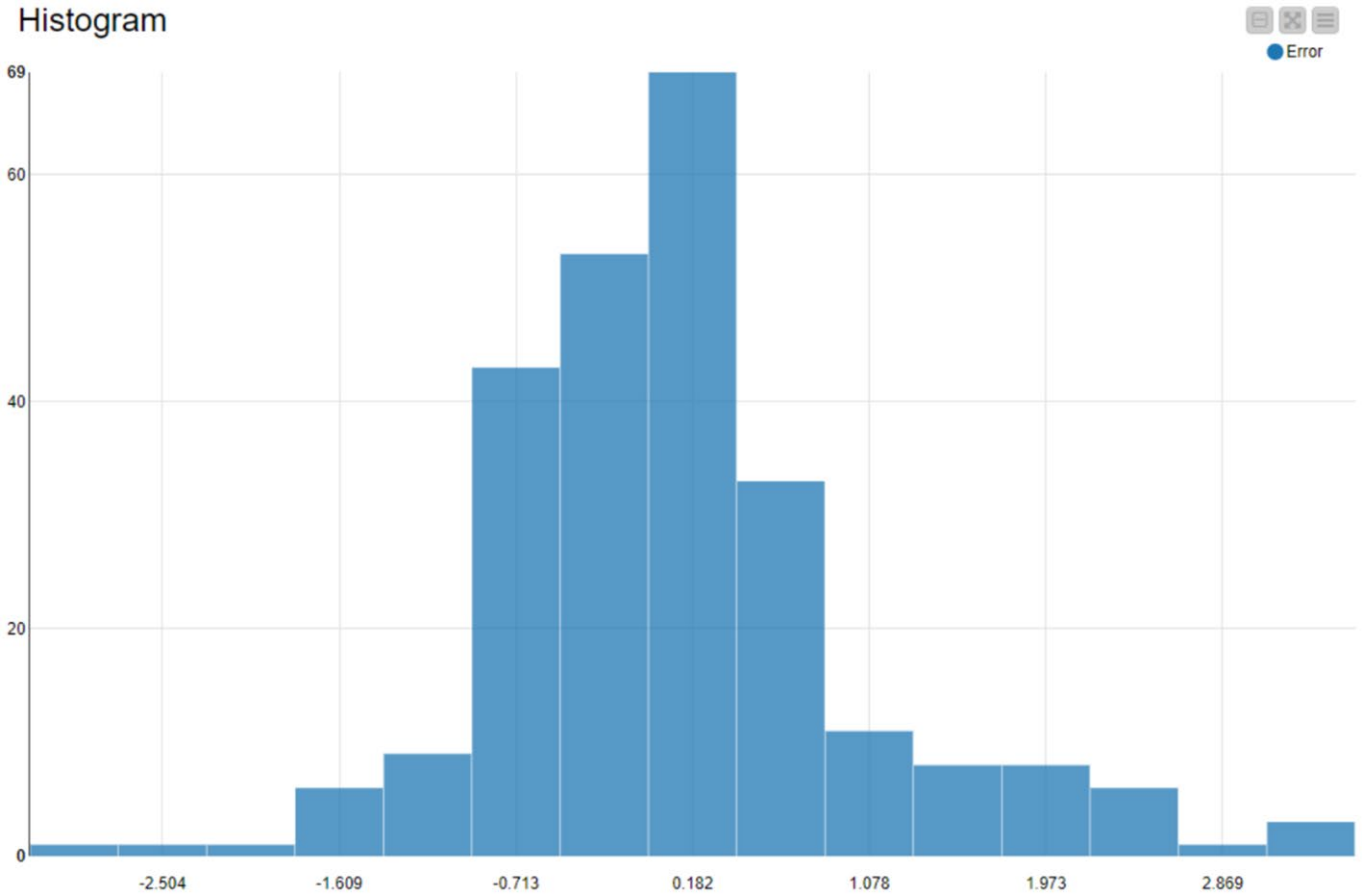
Error histogram.

The distribution is concentrated near zero, with the highest bars around a small positive error (≈ 0.18). This indicates the model’s typical errors are small, but the peak is slightly above 0, suggesting a mild tendency to overpredict on average (Figure 2).

Most spread (most errors) fall roughly within about [−1, +1], meaning that for the majority of country-year observations, the model’s predicted GDP per capita (in your scaled units) is close to the observed value.

The histogram is not perfectly symmetric: the right tail is longer than the left tail. Errors range from approximately +2.9 to about −2.5.

This indicates occasional large overpredictions (positive residual outliers) and fewer extreme underpredictions.

There are a small number of observations in the extreme bins on both sides (near −2.5 and +2.9), which are candidates for country-year outliers (structural economies, measurement differences, or unusual ICT/GDP combinations).

**Figure 3:**
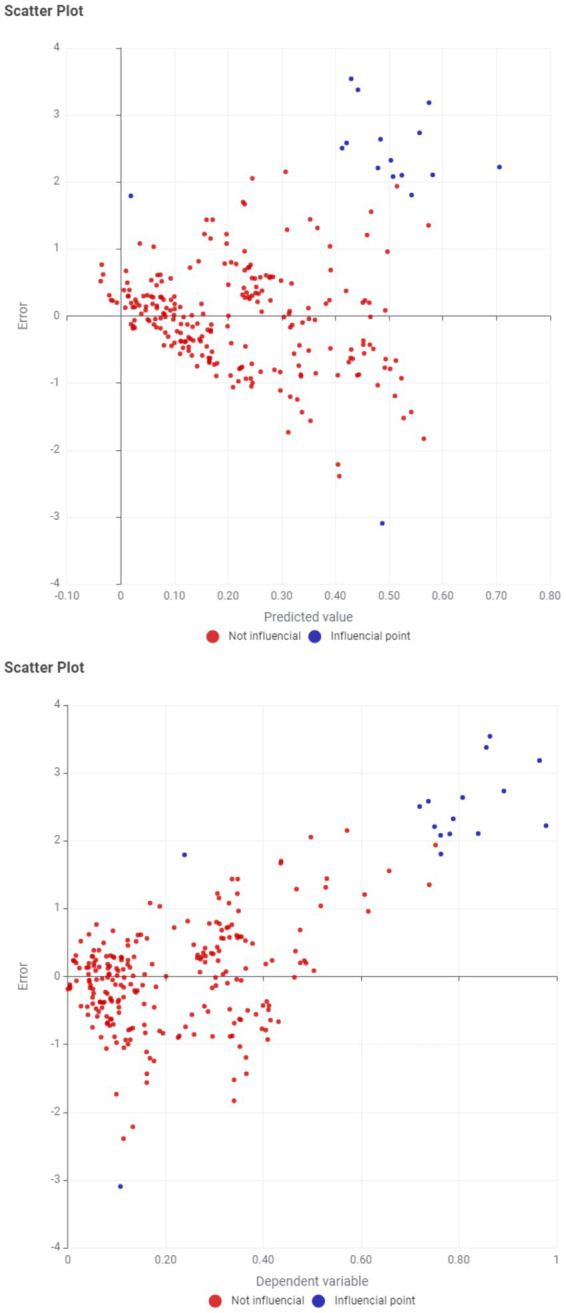
Error deviation.

Residual diagnostics (Figure 3) based on the residual-fitted relationship indicate two systematic features of the model. First, the residual patterns display country-specific structural bias (unobserved heterogeneity): the repeated underprediction for Luxembourg and Ireland (and, in several years, Norway) suggests the presence of persistent country-level factors not captured by the older adults ICT-use covariates, such as distinctive economic structures, multinational profit-shifting effects, resource-based income components, or cross-border commuting dynamics; in a panel-data context, these persistent differences are typically addressed through the inclusion of country fixed effects. Second, the dispersion of residuals increases for observations located in the upper and lower tails of the GDP-per-capita distribution, indicating heteroskedasticity (non-constant error variance); this motivates the use of heteroskedasticity-robust and/or clustered standard errors and, where appropriate, reconsideration of the dependent-variable functional form (e.g., logarithmic transformation) to stabilize variance.

The influence diagnostics further identify a small set of influential observations that concentrate on predictable country-year connections. Luxembourg is flagged repeatedly due to consistently large positive residuals (systematic underprediction), Ireland is similarly flagged across multiple years, and Norway appears influential in some years, plausibly reflecting high leverage associated with structural economic differences. In contrast, the Slovak Republic in 2022 is characterized by an extreme negative residual (substantial overprediction). These observations are influential because they combine unusually large residual magnitudes and/or high leverage relative to the remainder of the sample, and therefore have the potential to disproportionately affect coefficient estimates and inference if not explicitly accounted for in robustness checks.

The diagnostics support the conclusion that the model provides a reasonable fit for the central mass of EU country-year observations, where residuals are generally close to zero, but exhibits systematic bias at the extremes: it persistently underpredicts the highest-income economies (most notably Luxembourg and Ireland, and frequently Norway) and overpredicts lower-income cases (particularly the Slovak Republic over 2021–2023, with an especially pronounced deviation in 2022).

The concentration of influential observations in Luxembourg (2011, 2013, 2016–2017, 2019–2023, and 2020–2021) and Ireland (2022–2023) is economically consistent with the well-documented presence of multinational-driven distortions in GDP per capita in small open economies. In such cases, headline GDP per capita reflects profit shifting, intellectual property relocation, and accounting effects more than domestic productivity, while the ICT adoption profiles of people aged 55–74 evolve with social and institutional factors. Consequently, these observations create unusually large residuals and/or high leverage relative to the rest of the panel, thereby raising Cook’s distance and making them highly influential in coefficient estimation (Table 5).

**Table 5:** Cook distance for influential point.

Country	Year	Cook distance
Ireland	2023	0.011
Luxembourg	2023	0.017
Ireland	2022	0.011
Luxembourg	2022	0.012
Norway	2022	0.016
Slovak Republic	2022	0.017
Luxembourg	2021	0.021
Luxembourg	2020	0.012
Luxembourg	2019	0.014
Luxembourg	2017	0.015
Luxembourg	2016	0.018
Luxembourg	2013	0.013
Norway	2013	0.008
Italy	2011	0.010
Luxembourg	2011	0.018
Norway	2011	0.011

Norway appears influential in 2011 and 2022 (and close to the threshold in 2013), which is economically plausible given the role of resource rents and terms-of-trade effects in GDP per capita, which are not directly captured by household ICT usage variables. This implies that Norway’s income level can be structurally high relative to its older adults digital-use profile, again producing leverage in the regression. The Slovak Republic in 2022 is influential in the opposite direction: the model substantially overpredicts GDP per capita given its ICT indicators, suggesting a disconnect between measured digital participation and income generation, potentially due to sectoral composition, productivity constraints, or post-shock adjustment dynamics.

These findings imply that the estimated ICT-income relationships are broadly stable for the majority of EU country-years, but inference can be sensitive to a small number of structurally atypical economies.

Few variables indicate severe multicollinearity, typically causing unstable coefficient signs, inflated standard errors, and sensitivity to small changes in model specification (Table 6).

**Table 6:** Multicollinearity test.

Column header	VIF values
Lack of skills for submitting forms	1.391
Rare using	25.020 (consider eliminating)
Subscriptions Fixed broadband	1.180
Daily using	39.759 (consider eliminating)
Social networking	5.045
Online course	3.595
Online ICT services	2.784
Seeking health information	5.541
Online purchasing	2.939
GDP per capita	3.468

These variables are not strongly explained by the remaining regressors, and their coefficients should be comparatively stable with respect to multicollinearity.

VIFs fall into three practical categories:

5 variables have no multicollinearity concern (VIF < 5) [Subscriptions Fixed broadband: 1.18; Lack of skills for submitting forms: 1.39; Online ICT services: 2.78; GDP per capita: 3.47; Online course: 3.59; Online purchasing (2.939)].

2 variables have Moderate multicollinearity (5 ≤ VIF < 10) (Social networking: 5.04; Seeking health information: 5.54). Some overlap exists, but the coefficients are not much bigger than 5. It has a negligible impact on the model and can be used for interpretation.

In this research, the multicollinearity threshold value is set to 10 (50, 51). Because of that, the variables of Internet use [Rarely used (25.02) and daily used (39.76)] are highly redundant with other variables in your model. In practice, they are likely capturing the same underlying latent construct (overall internet adoption/intensity and digitally embedded behavior) and are strongly correlated with the other usage indicators.

This finding is consistent with the earlier regression table, where Daily Internet use and Rare Internet use were not significant, an expected outcome when they largely duplicate information already contained in other usage measures.

Considering all the previous analyses, the final model follows Equation 4:


$$\begin{array}{c} \mathrm{GDP}\;\mathrm{per}\;\text{capita}=0.208*\text{Lack of skills for submitting forms}\\ -0.1983*\text{Subscriptions Fixed broadband}\\ -0.221*\text{Social networking}+0.447*\text{Online course}\\ +0.1115*\text{Online}\;\mathrm{ICT}\;\text{services}\\ -0.461*\text{Seeking health information}\\ +0.770*\text{Online purchasing}+0.035 \end{array}$$ (4)

Given the large positive coefficient for online purchasing (*β* = 0.770), the results indicate that countries with higher shares of people aged 55–74 who purchase online tend to have higher GDP per capita, conditional on other ICT indicators. Together, this variety of skills ack (in our research for submitting forms, but we can transpose it to all skills; *β* = 0.208) supports interventions that reduce barriers to digital transactions, including strengthened consumer protection and anti-fraud measures, improved usability of electronic identification and authentication, and support for enterprises to adopt e-commerce capabilities.

The second-largest positive association is observed for online course participation (*β* = 0.447, implying that lifelong learning and digital skills upgrading among people aged 55–74 are systematically associated with higher GDP per capita. This finding appears to emphasize the importance of including subsidized or co-financed digital training and course designs tailored to older learners.

Use of online ICT services is also positively associated with GDP per capita (*β* = 0.1115), suggesting that improving the accessibility and usability of public and private online services for people aged 55–74 may complement broader digitalization and income outcomes.

Coefficients with negative signs for social networking (*β* = −0.221) and seeking health information online (*β* = −0.461) suggest a lack of communication and knowledge among people aged 55–74. Comparison of transactional and learning-oriented behaviors with lack of skills and social networking, these activities are less closely aligned with higher GDP per capita.

To assess whether the estimated relationships are robust to unobserved country-specific heterogeneity, an additional panel specification with country fixed effects was estimated (Table 7). While the baseline model (Equation 4) captures main variation, the fixed-effects model demonstrates differences across countries.

**Table 7:** Linear regression results with fixed effects.

Variable	Coeff.	Std. err.	t-value	P > |t|
Lack of skills	0.01	0.03	0.50	0.62
Rare using	0.05	0.07	0.64	0.52
Subscriptions Fixed broadband	−0.04	0.04	−1.01	0.31
Daily using	0.07	0.10	0.66	0.51
Social networking	−0.07	0.04	−1.63	0.11
Online course	0.16	0.04	4.44	0.00
Online ICT services	0.04	0.04	1.12	0.27
Seeking health information	−0.16	0.04	−3.81	0.00
Online purchasing	0.17	0.07	2.25	0.03
Country Name = Belgium	−0.03	0.02	−1.58	0.12
Country Name = Bulgaria	−0.24	0.03	−9.24	0.00
Country Name = Croatia	−0.21	0.02	−8.75	0.00
Country Name = Czechia	−0.17	0.02	−9.09	0.00
Country Name = Denmark	0.05	0.03	1.85	0.07
Country Name = Estonia	−0.20	0.02	−9.55	0.00
Country Name = Finland	−0.02	0.02	−0.91	0.37
Country Name = France	−0.09	0.03	−3.64	0.00
Country Name = Germany	−0.04	0.02	−1.59	0.11
Country Name = Greece	−0.17	0.03	−6.72	0.00
Country Name = Hungary	−0.20	0.02	−8.47	0.00
Country Name = Iceland	0.04	0.03	1.11	0.27
Country Name = Ireland	0.18	0.02	9.27	0.00
Country Name = Italy	−0.09	0.03	−2.73	0.01
Country Name = Latvia	−0.22	0.02	−9.44	0.00
Country Name = Lithuania	−0.18	0.02	−7.83	0.00
Country Name = Luxembourg	0.47	0.02	21.14	0.00
Country Name = Netherlands	−0.02	0.02	−0.92	0.36
Country Name = Norway	0.22	0.02	9.25	0.00
Country Name = Poland	−0.23	0.02	−11.61	0.00
Country Name = Portugal	−0.17	0.03	−6.15	0.00
Country Name = Romania	−0.23	0.03	−8.95	0.00
Country Name = Slovak Republic	−0.22	0.02	−11.67	0.00
Country Name = Slovenia	−0.17	0.02	−8.20	0.00
Country Name = Spain	−0.16	0.02	−7.57	0.00
Country Name = Sweden	−0.04	0.02	−1.53	0.13
Intercept	0.28	0.02	11.58	0.00

The comparison of the two specifications reveals that several variables that were statistically significant in the pooled model lose significance once country fixed effects are included, indicating that part of the baseline relationships is driven by persistent cross-country differences. In contrast, online course participation, online purchasing, and seeking health information remain statistically significant, suggesting that these variables have robust within-country associations with GDP per capita. Austria was chosen and basic country for comparison.

Luxembourg has the largest positive fixed effect at about 0.47, which indicates the largest upward country-specific deviation relative to the reference country, after controlling for observed ICT variables.

The next strongest positive country effects are found for Norway (*β* = 0.22, *p* < 0.001) and Ireland (*β* = 0.18, *p* < 0.001), which also suggests that these economies have persistent structural advantages not captured by the digital indicators. In Ireland, this may reflect multinational accounting effects and foreign-investment-driven GDP distortions, while in Norway it is consistent with resource-based income effects.

Countries with small positive but statistically non-significant effects, suggest the evidence is not strong enough to conclude that the difference is statistically reliable. It is Denmark (*β* = 0.05, *p* = 0.07) and Iceland (*β* = 0.04, *p* = 0.27).

Countries such as Belgium, Germany, Finland, Netherlands, and Sweden have coefficients close to zero and statistically non-significant, meaning their unexplained country-specific deviation is relatively modest compared with the reference category.

The fixed-effects results confirm that digital engagement explains part of income variation within countries, but also highlight the importance of structural country-specific factors that are not captured by ICT indicators alone.

## 5 Discussion

This study’s results refine how the Silver Economy-digitalization link should be interpreted at the macro level. Rather than treating the 55–74 age group’s country-level internet-use profile as a uniform phenomenon, the pattern of coefficients indicates that different online activities likely capture different underlying social, institutional, and economic mechanisms. In particular, transactional and learning-oriented behaviors appear to function as system-level markers of digital ecosystem maturity, where trust, consumer protection, service design, and institutional capacity enable wider participation beyond basic connectivity at the country level. This supports the broader literature arguing that digitalization produces uneven benefits and that inclusion depends on both skills and enabling environments, otherwise leading to exclusion or “adverse incorporation.”

Several associations underline the importance of context and measurement. The positive sign for reported lack of skills in submitting forms is best understood as an exposure/reporting effect: where e-government is widespread, barriers become more visible and are more often reported. Similarly, the negative partial association for health information search is consistent with a need-driven interpretation: higher search activity may reflect healthcare access constraints, greater health burden, or substitution for formal pathways once other forms of digital participation are controlled for. The negative sign for social networking suggests that, conditional on economically embedded uses, social-platform activity may capture a different digital profile, one oriented toward communication rather than transactions or structured skill upgrading without implying lower social value.

Diagnostics indicate that the interpretation must account for the model structure. High VIF values confirm that frequency measures (“rare” and “daily” use) overlap strongly with activity-based indicators; once purposes are included, intensity largely loses distinct informational content. The infrastructure proxy (fixed broadband subscriptions) illustrates how upstream variables can change sign in multivariate settings, especially when measured as absolute counts rather than per-capita rates. The year-level deterioration in accuracy in 2022 plausibly reflects macro shocks that decouple short-run income dynamics from household ICT profiles. Finally, influential cases (Luxembourg, Ireland, and, in some years, Norway) signal that GDP per capita can be structurally distorted by factors orthogonal to people aged 55–74′ digital behavior, motivating robustness checks and alternative outcome measures. These considerations point to the next research steps: fixed-effects panel models, alternative income indicators (e.g., GNI), per-capita infrastructure scaling, and composite indices of “productive engagement” to reduce redundancy and improve interpretability.

## 6 Conclusion

This study examined country-level ICT-use indicators for people aged 55–74 and shows that digital engagement in later life aligns with cross-country differences in income levels, with the models explaining a substantial share of variation in GDP per capita and GNI per capita. However, the observed relationships are associational and do not establish that digital engagement causes changes in GDP per capita (or vice versa). Importantly, the results indicate that the economic relevance of digital inclusion is not captured by internet access or frequency of use alone, but by the composition of online activities. Across EU countries, the strongest positive associations with GDP per capita were observed for online purchasing and online course participation, suggesting that transactional participation and lifelong learning are the most development-relevant forms of digital engagement in the silver economy. In contrast, online ICT services contribute to a lesser extent. In contrast, general intensity measures (rare/daily use) do not add explanatory power once activity types are included, consistent with their severe multicollinearity (VIF > 10).

The comparison between the GDP and GNI models shows that several relationships are stable across both outcome indicators, particularly for online purchasing, Seeking health information, social networking, Lack of skills, and Fixed broadband subscriptions. This indicates that these associations are not driven solely by the use of GDP per capita and remain visible when development is measured by resident income instead of domestic output.

At the same time, some variables are clearly sensitive to the choice of dependent variable. The most important example is online course participation, which is one of the strongest predictors in the GDP model but becomes entirely irrelevant in the GNI model. Online ICT services also lose significance when switching to GNI, while Daily using becomes stronger. These differences imply that some digital behaviors are associated more closely with production-oriented development, whereas others are more related to income conditions at the resident level.

Theoretically, these findings contribute to Silver Economy and digital inclusion research by empirically supporting a shift from “connectivity/intensity” toward “productive digital engagement” as the key development-relevant dimension of country-level digital engagement profiles among people aged 55–74. This is consistent with conceptual work arguing that digitalization generates unequal benefits unless it enables meaningful participation and capability use (10), and it refines “ICT–growth” interpretations discussed in the productivity-paradox literature by showing that not all digital use is equally aligned with economic outcomes (25). Our results extend existing evidence on aging consumers and e-commerce by demonstrating, at the macro-EU level, that e-commerce adoption among people aged 55–74 is strongly associated with higher income contexts (27). Similarly, the positive association between online course participation and active aging, and the human-capital mechanisms emphasized in Silver Economy scholarship (6, 52, 53), aligns with research suggesting that capability upgrading can mitigate aging-related productivity constraints (26). At the same time, negative partial associations for health information search and social networking are compatible with the digital divide and digital health literature, which stresses that some online behaviors may be need-driven or less economically embedded once transactional and learning uses are accounted for (11, 18, 54, 55).

Model diagnostics further show that inference is sensitive to a small set of structurally atypical economies (e.g., Luxembourg, Ireland, and in some years Norway), and that macroeconomic disruption weakens the contemporaneous mapping between ICT behaviors and GDP per capita. Overall, the results motivate prioritizing interventions that increase the national prevalence of higher-value digital participation among people aged 55–74, particularly in e-commerce and lifelong learning, complemented by efforts to improve the accessibility and usability of essential online services.

The country fixed effects show that the digital-behavior variables explain only part of the variation in GDP per capita. The remaining country coefficients capture deep structural differences that are not directly measured in the model, such as production structure, macroeconomic specialization, institutional quality, innovation systems, labor productivity, external dependency, or GDP accounting characteristics. The fixed-effects specification demonstrates that part of the variation in the pooled model was driven by country-specific characteristics rather than by ICT-use indicators alone.

## Data Availability

The original contributions presented in the study are included in the article/supplementary material, further inquiries can be directed to the corresponding author.
